# Cardiac glycosides in patients with heart failure and reduced ejection fraction: a systematic review and meta-analysis

**DOI:** 10.3389/fcvm.2026.1746467

**Published:** 2026-03-13

**Authors:** Dayang Wang, Xiaoqing Xu, Jingyao Lu, Jiayi Gao, Yingyu Li, Guozhong Pan, Wenhua Peng

**Affiliations:** 1 Institute of Cardiovascular Diseases, Dongzhimen Hospital, Beijing University of Chinese Medicine, Beijing, China; 2 Second Department of Cardiology, Dongzhimen Hospital, Beijing University of Chinese Medicine, Beijing, China; 3 Beijing Hepingli Hospital, Beijing, China

**Keywords:** cardiac glycosides, digitoxin, digoxin, heart failure and reduced ejection fraction, meta-analysis, prognosis

## Abstract

**Background:**

The prognostic benefits of cardiac glycosides in patients with heart failure and reduced ejection fraction (HFrEF) remain controversial. Although digitoxin exhibits more favorable pharmacokinetic characteristics than digoxin, its potential to provide superior clinical efficacy has not yet been established.

**Methods:**

A systematic review and meta-analysis were performed. The PubMed, EMBASE, Cochrane Library, and Web of Science databases were systematically searched for relevant studies published up to September 12, 2025. The study population was limited to patients with HFrEF. Randomized controlled trails that compare clinical outcomes between cardiac glycosides and placebo were included. The primary outcome was all-cause mortality, and the secondary outcome was hospitalization due to heart failure. Additionally, an indirect comparison was performed to compare the efficacy of digitoxin vs. digoxin. This study was registered with PROSPERO (No. CRD420251142262).

**Results:**

A total of 6 RCTs encompassing 8,488 patients were included. Compared with placebo, cardiac glycoside therapy was associated with a significantly lower risk of hospitalization for heart failure [HR = 0.79, 95% CI (0.67–0.94)]. However, there was no significant difference in all-cause mortality [HR = 0.98, 95% CI (0.92–1.04)]. Furthermore, no significant differences were observed between digitoxin and digoxin in terms of mortality reduction [HR = 0.93, 95% CI (0.77–1.13)] or hospitalization risk for heart failure [HR = 1.35, 95% CI (0.70–2.60)].

**Conclusion:**

In patients with HFrEF, cardiac glycoside therapy may reduce the risk of hospitalization for heart failure. Compared with digoxin, digitoxin has not demonstrated any significant advantage with respect to all-cause mortality or hospitalization for heart failure.

**Systematic Review Registration:**

https://www.crd.york.ac.uk/PROSPERO/view/CRD420251142262, PROSPERO CRD420251142262.

## GRAPHICAL ABSTRACT

**Figure:**
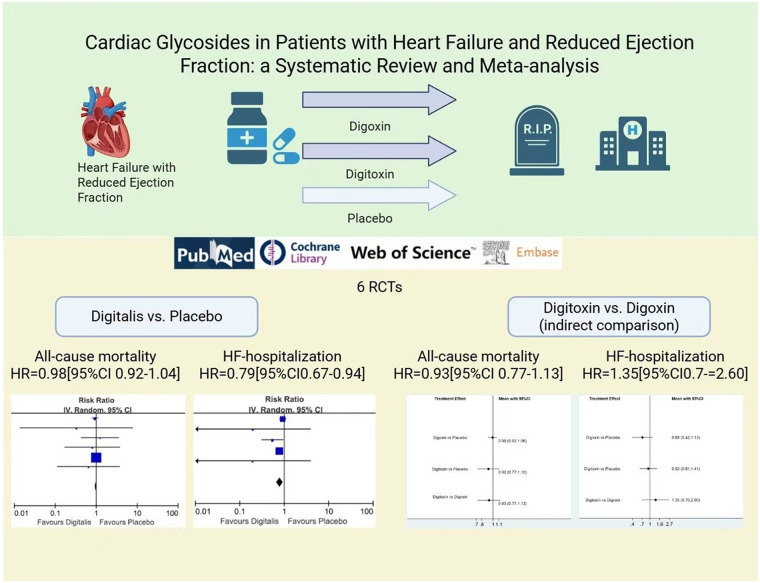

## 1 Introduction

Cardiac glycosides have been employed in the treatment of heart failure for over two centuries. Cardiac glycosides enhance myocardial contractility by inhibiting Na^+^-K^+^-ATPase, which increases the intracellular calcium concentration in both cardiomyocytes and the sarcoplasmic reticulum. Concurrently, they reduce heart rate through indirect suppression of sympathetic tone (1). The aforementioned mechanism can effectively alleviate symptoms of fatigue and dyspnea caused by impaired cardiac contractility. However, the role of cardiac glycosides in improving the prognosis of heart failure with reduced ejection fraction (HFrEF) remains controversial. For instance, the Digitalis Investigation Group (DIG) trial did not demonstrate a mortality benefit with digoxin in heart failure patients (2). Consequently, the recommendations of current clinical guidelines (3) is that cardiac glycosides alleviate symptoms in patients with heart failure but do not improve prognosis.

Digitoxin is another type of cardiac glycoside distinct from digoxin (4, 5). Despite sharing a common mechanism, digitoxin and digoxin have distinct pharmacokinetic profiles regarding absorption, metabolism, and bioavailability. The DIGIT-HF trial demonstrated that digitoxin significantly reduced composite outcome of all-cause mortality and heart failure (HF) rehospitalization rates in patients with HFrEF (6). The outcome of DIGIT-HF trial has challenged the conventional understanding of cardiac glycosides in the treatment of HFrEF. Therefore, we conducted a systematic review and meta-analysis of current randomized controlled trials (RCTs) on digitalis therapy, aiming at evaluating the efficacy of cardiac glycosides in improving HFrEF prognosis and determining whether a differential effect exists between digitoxin and digoxin.

## 2 Methods

The protocol of this study was registered with PROSPERO (NO. CRD420251142262). The study reported in this article adhered to the Preferred Reporting Items for a Systematic Review and Meta-analysis (PRISMA) 2020 update (7).

### 2.1 Data sources and searches

The literature searches were conducted in the following four databases: PubMed, EMBASE, Cochrane Library and Web of Science. The publication time was set from the inception to September 12th, 2025. We used the following subject terms and free words to perform search: “cardiac glycosides”, “heart failure with reduced ejection fraction”, “digitoxin”, “mortality”, “rehospitalization” etc. Supplementary Table S1 showed search strategies in detail.

### 2.2 Eligibility criteria, study selection and data extraction

Studies were considered eligible for inclusion if they met following criterion: 1.were published as a full-length article; 2.were written in English; 3.study type was RCT; 4.included comparisons between cardiac glycosides (digoxin or digitoxin) combined with guideline directed medical treatment (GDMT) vs. placebo combined with GDMT; 5.reported raw data of case number of all-cause mortality or HF rehospitalization.

Two independent reviewers (DW and XX) scanned titles and abstracts according to the inclusion criteria, reviewed full-text articles, and determined their eligibility. Any discrepancy regarding searches and selection was discussed in consultation with and resolved by a third reviewer (WP). The full text was retrieved for further inspection if a study potentially met the inclusion criteria.

Two reviewers independently conducted data extraction. The data included: 1. study-level general information; 2. baseline characteristic of population; 3. outcomes from original eligible sources, including all-cause mortality or HF rehospitalization. The ascertainment of clinical events was accepted as reported. Discrepancy, if any, was verified and resolved by a third reviewer (WP). Records of studies were managed with the Endnote X9 software.

### 2.3 Quality assessment

The methodological Quality of RCTs was assessed using the “Revised Cochrane Risk of Bias Tool for Randomized Trials” (RoB-2) (8).

### 2.4 Statistical analysis

Meta-analyses were conducted for comparable studies using RevMan 5.4.1 software. Summary effect size for all independent events was calculated using pooled hazard ration (HR). Data presented in form of the number of cases were converted to HR and 95% confidence interval (95%CI) utilizing the embedded calculation program within software. A random-effects model was used in all the outcomes. We did not conduct pooled analyses for outcomes reported in fewer than three studies. Inter-study heterogeneity was assessed using the I2 statistic, which was defined as I2 values of 50% or greater. The z statistic was computed for each outcome of interest, and the results were considered statistically significant at 1-sided *p* < 0.05. Meta-analysis results were presented using forest plots.

To evaluate robustness, we performed a sensitivity analysis involving leave-one-out method to ascertain their impact on the total results.

A network meta-analysis was performed to assess the effectiveness of the treatments. Because the study included only three treatment options (digoxin, digitoxin, and placebo), placebo was used as the reference node to enable indirect comparisons between digitoxin and digoxin regarding all-cause mortality and HF hospitalization. Consistency assessment was not conducted because all data originated from indirect treatment comparisons, and no head-to-head RCTs were available. Indirect comparisons were implemented using the “mvmeta” and “network” packages in STATA 17 software.

The publication bias was assessed using funnel plots by displaying individual study effect for the outcomes of interest. Funnel plot asymmetry was also evaluated using Egger's test (with 1-sided *p* < 0.1 indicating significant publication bias). The publication bias assessments were conducted using STATA 17 software.

## 3 Results

### 3.1 Study selection and characteristics

**Figure 1:**
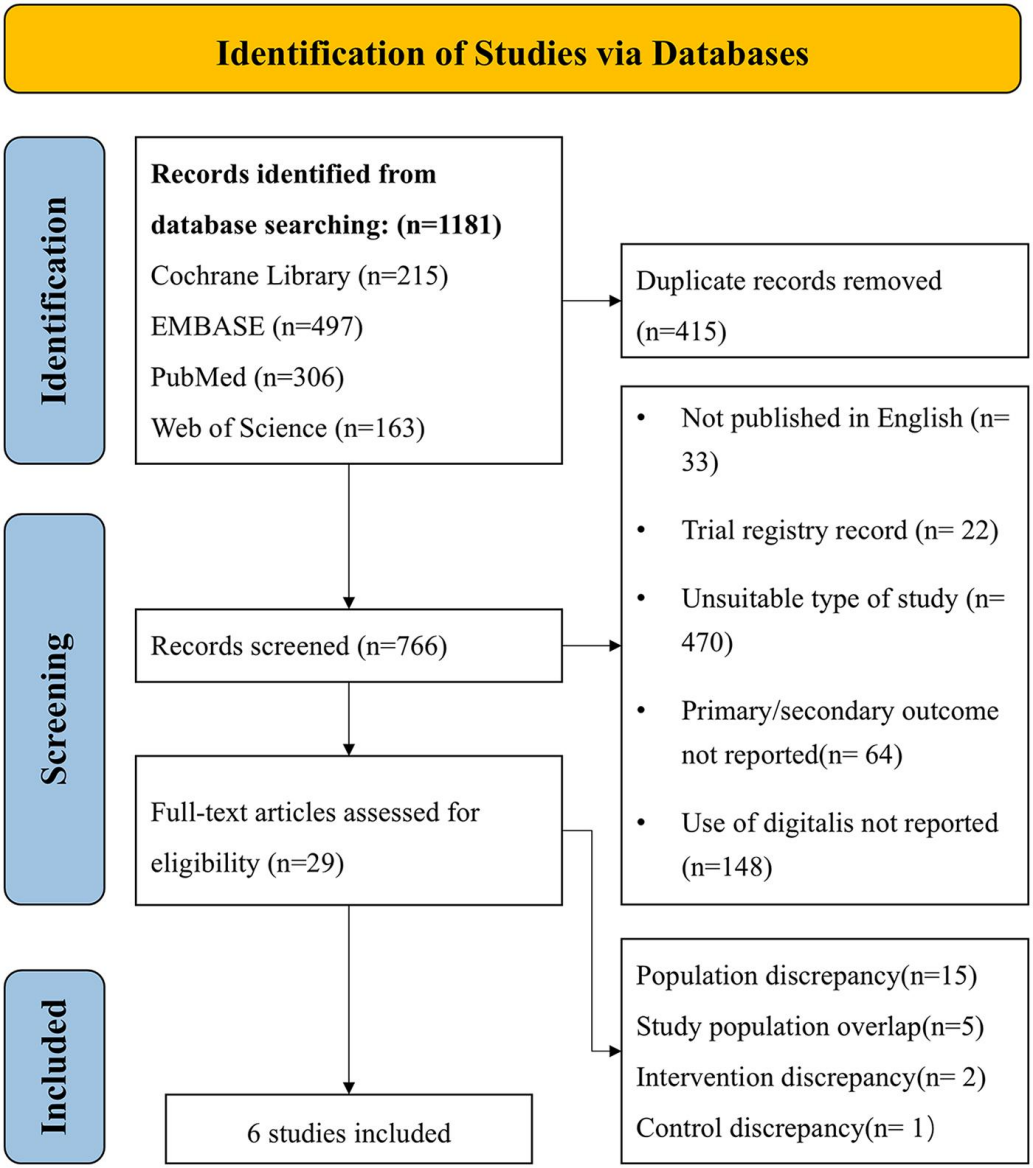
Flow chart of study selection process.

From the initial search, we identified 1,181 studies, of which 415 duplicates were removed. Of the 766 remaining records, 737 were excluded after screening titles and abstracts due to inappropriate article types, inappropriate language, irrelevance with digitalis, or primary/secondary outcome not reported. After assessing the full text of the remaining 29 articles, we excluded 15 for irrelevant population, 1 for irrelevant control, 2 for irrelevant intervention, and 5 for Study population overlap. Ultimately, 6 studies met the inclusion criteria and were included in the systematic review. Flow chart of the study selection process is shown in Figure 1.

A total of 8,488 patients from 6 RCTs were included. The duration of follow-up ranged from 6 months to 4 years. Among the cardiac glycoside interventions, five studies investigated digoxin, and one study investigated digitoxin. The general characteristics of the included studies are summarized in Table 1. The mean age of participants ranged from 56.8 to 65.9 years. The proportion of female participants ranged from 17.1% to 75.7%, and the mean left ventricular ejection fraction (LVEF) was less than 30% across all studies. The baseline characteristics of the included studies are presented in Table 2.

**Table 1:** General characteristic of included studies.

Study ID	Country	Number of patients	Follow-up period (month)	Reported outcomes	Drug	LVEF threshold
Captopril-Dig Group 1988	United States, Canada and New Zealand	196	6	①②	digoxin	40
DIBianco 1989	United States and Canada	111	3	①	digoxin	40
Blackwood 1990	England	61	3	①②	digoxin	40
Van Veldhuisen 1993(DIMT)	Netherlands	108	6	①②	digoxin	45
Digitalis Investigation Group 1997 (DIG)	United States and Canada	6,800	37	①②	digoxin	45
Bavendiek 2025 (DIGIT-HF)	Austria, Germany, and Serbia	1,212	48	①②③	digitoxin	40 for NYHA III IV, 30 for NYHA II

Outcomes Reported: ① all-cause mortality; ② heart failure rehospitalization; ③ Composite of all-cause mortality or heart failure rehospitalization.

LVEF = left ventricular ejection fraction.

**Table 2:** Baseline characteristics of included studies.

Study ID	Age, year	Sex, female%	LVEF%	Atrial fibrillation	Baseline heart rate	EGFR	GDMT			
							ACEI/ARB	BB	MRA	SGLT2i
Captopril-Dig Group 1988	57.8	17.1	25.2	NR	NR	NR	–	–	87	–
DIBianco 1989	60.0	75.7	25.0	NR	NR	0–40: 14.88% 40–80: 56.76% > 80: 27.90%	25.4%	–	–	–
Blackwood 1990	60.0	49.6	NR	NR	NR	NR	–	–	–	–
Van Veldhuisen 1993(DIMT)	61.0	13.9	29.0	NR	77.5	NR	–	–	–	–
Digitalis Investigation Group 1997 (DIG)	63.5	22.4	29.8	NR	NR	NR	94.5%	–	–	–
Bavendiek 2025 (DIGIT-HF)	65.9	20.4	28.6	27.2	73.9	65.1	35.9%	95.7%	76.2%	19.3%

### 3.2 Quality assessment

**Figure 2:**
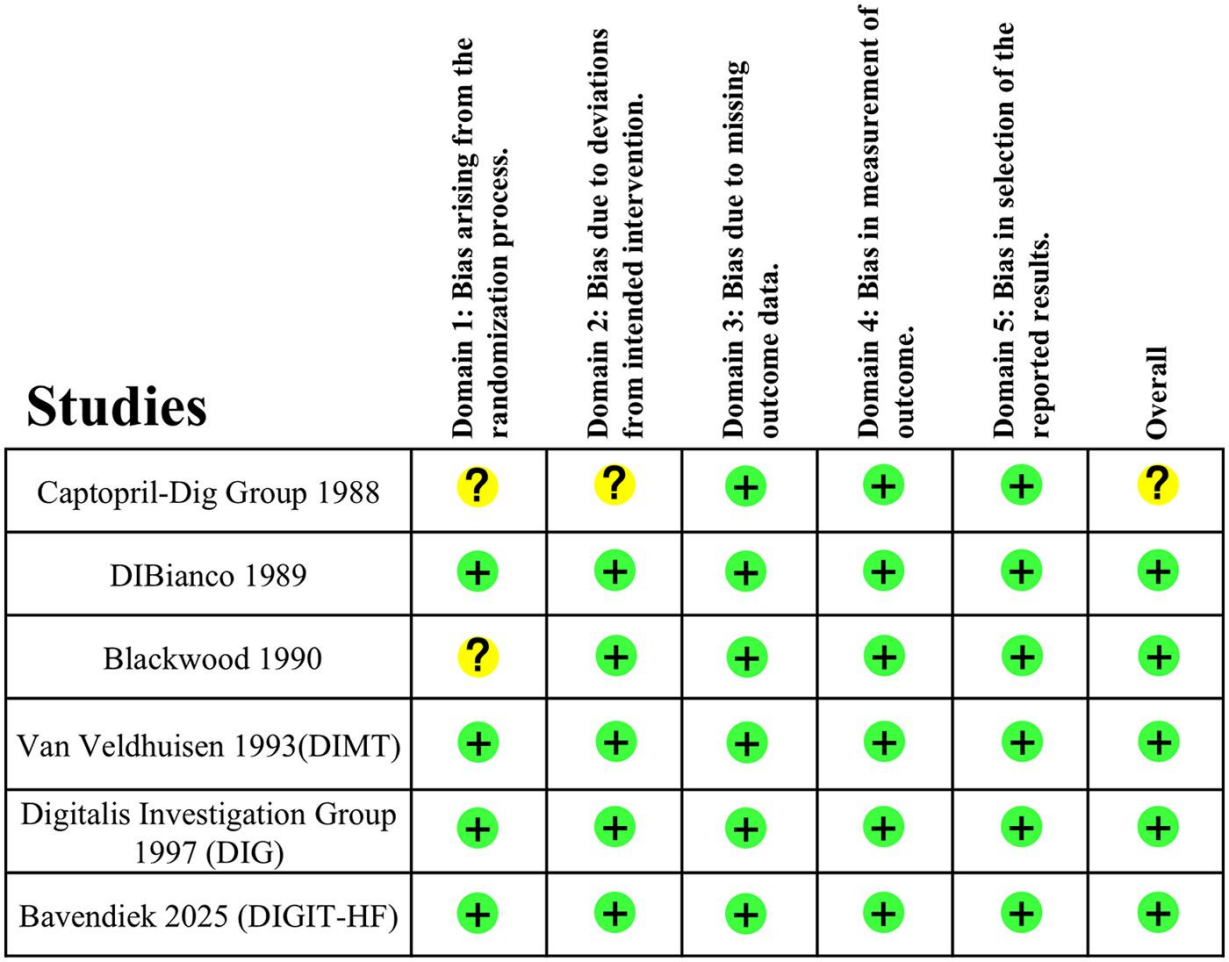
Risk of bias assessed by RoB-2 tool.

The 6 RCTs included in our meta-analysis were regarded to have “low risk of bias”. Details of quality assessment for individual studies using RoB-2 tools are shown in Figure 2.

### 3.3 Outcomes

#### 3.3.1 Meta-analysis of cardiac glycosides in patients with HFrEF

**Figure 3:**
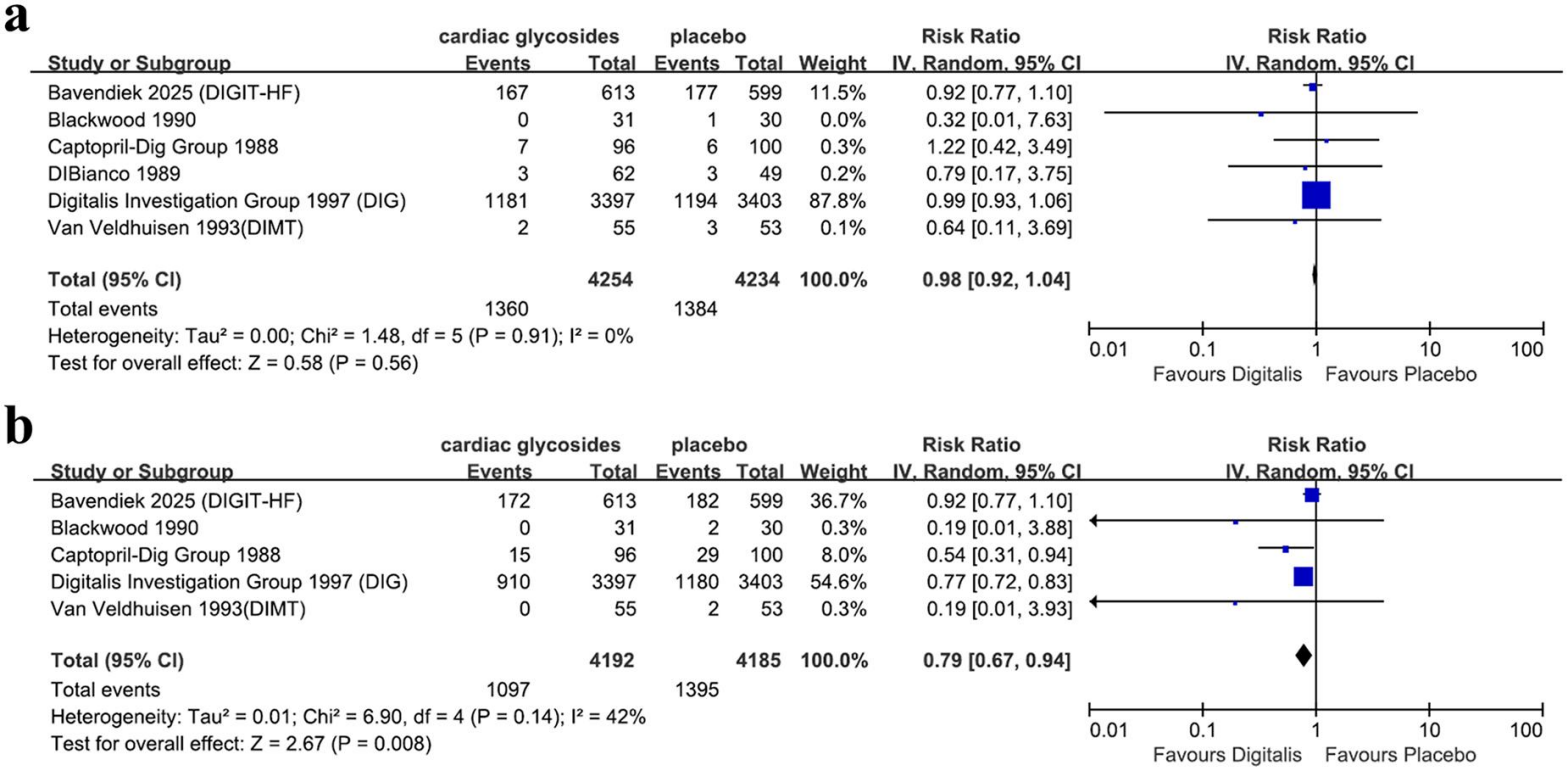
Forest plot of **(a)** all-cause mortality and **(b)** HF hospitalization of HFrEF patients with digitalis + GDMT vs. GDMT.

The outcome of all-cause mortality was reported in 6 studies. Compared with the placebo group, treatment with cardiac glycosides was not associated with a statistically significant difference in all-cause mortality (HR = 0.98, 95% CI 0.92–1.04; Figure 3a). The outcome of HF hospitalization was reported in five studies. Compared with the placebo group, cardiac glycoside therapy was associated with a significantly lower risk of hospitalization for heart failure (HR = 0.79, 95% CI 0.67–0.94; Figure 3b).

#### 3.3.2 Indirect comparison between digoxin and digitoxin

**Figure 4:**
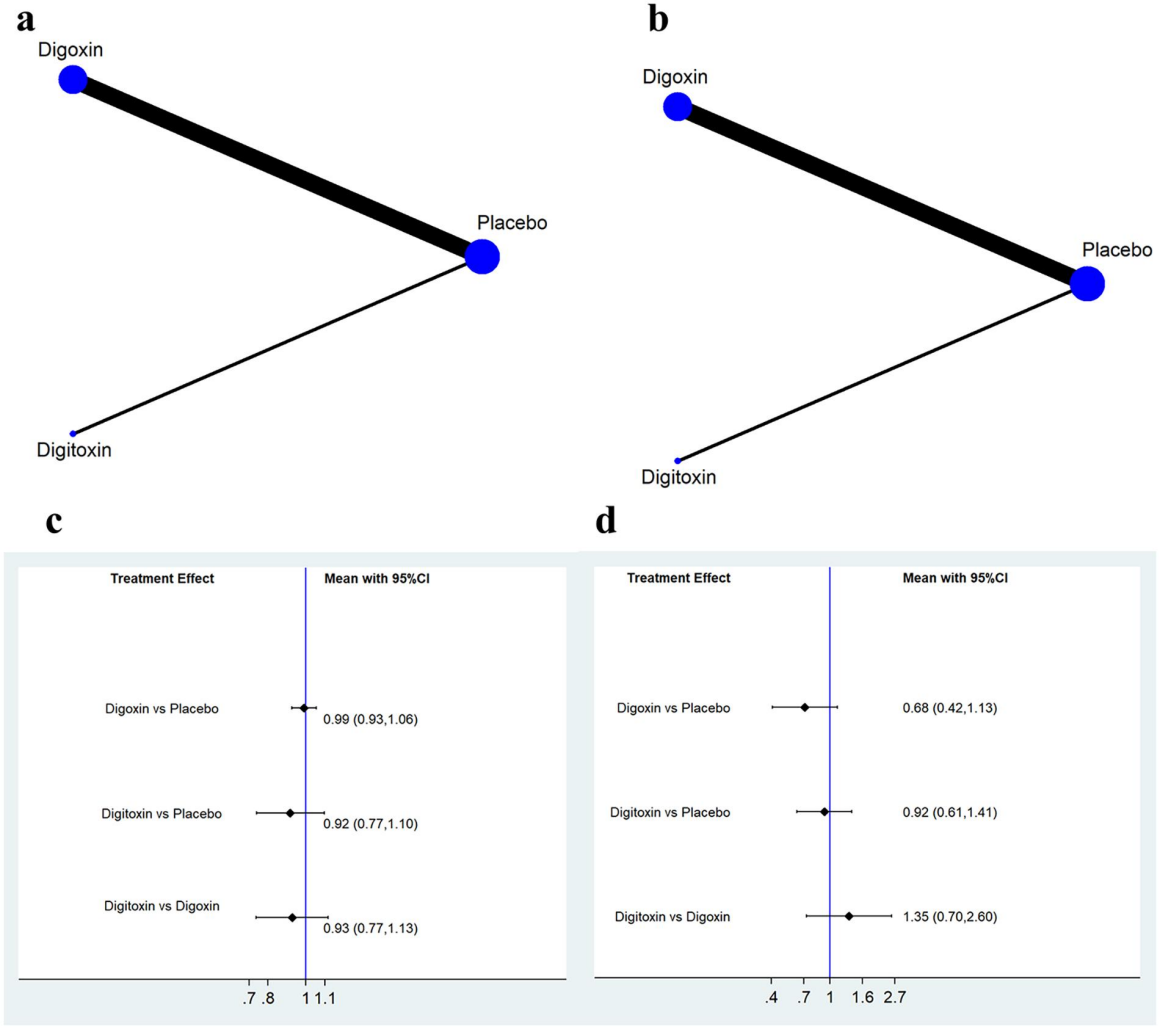
Indirect comparison between digitoxin and digoxin. **(a)** Network plot for all-cause mortality; **(b)** Network plot for hospitalization due to heart failure; **(c)** Forest plot illustrating all-cause mortality; **(d)** Forest plot illustrating hospitalization due to heart failure.

Using placebo control as a “reference node”, we conducted an indirect comparison of digitoxin and digoxin regarding clinical outcomes (Figures 4a, 4b). No significant differences were found between digitoxin and digoxin in all-cause mortality (HR = 0.93, 95% CI 0.77–1.13; Figure 4c) or risk of hospitalization for heart failure (HR = 1.35, 95% CI 0.70–2.60; Figure 4d).

### 3.4 Publication bias and sensitivity analysis

The funnel plot for all-cause mortality exhibited asymmetry (Supplementary Figure S1). Egger's regression test did not detect evidence of small-study effects or publication bias (*p* = 0.204, Supplementary Figure S2). We performed leave-one-out sensitivity analyses for all-cause mortality and HF hospitalization. The pooled results were generally robust; however, the HF hospitalization outcome was sensitive to exclusion 1 study (DIG trial), which altered the pooled estimate (Table 3).

**Table 3:** Sensitivity analysis for comparison of cardiac glycosides vs. placebo.

Study omitted	Estimate hazard ratio and 95%Confidence Interval
All-cause mortality	**0.98 [0.92, 1.04]**
Captopril-Dig Group 1988	0.98 [0.92, 1.04]
DIBianco 1989	0.98 [0.92, 1.04]
Blackwood 1990	0.98 [0.92, 1.04]
Van Veldhuisen 1993(DIMT)	0.98 [0.92, 1.04]
Digitalis Investigation Group 1997 (DIG)	0.92 [0.77, 1.10]
Bavendiek 2025 (DIGIT-HF)	0.99 [0.93, 1.06]
HF hospitalization	**0.79 [0.67, 0.94]**
Captopril-Dig Group 1988	0.82 [0.70, 0.96]
Blackwood 1990	0.80 [0.67, 0.95]
Van Veldhuisen 1993(DIMT)	0.80 [0.67, 0.95]
Digitalis Investigation Group 1997 (DIG)	0.71 [0.44, 1.15]*
Bavendiek 2025 (DIGIT-HF)	0.74 [0.62, 0.88]

HF, heart failure.

The bold values indicate the key computational results used.

* Omitting the study affects the stability of total results.

## 4 Discussion

### 4.1 Main findings

We aimed to further clarify the impact of cardiac glycosides on the prognosis of patients with HFrEF and to determine the efficacy differences between digitoxin and digoxin through a network meta-analysis. Based on the 6 RCTs included in our study, we found that cardiac glycosides may not be associated with lower risk of all-cause mortality in HFrEF patients but reduced the rate of HF hospitalization. No significant differences were observed between digitoxin and digoxin in terms of mortality reduction or HF hospitalization.

### 4.2 Interpretation

Among the 6 RCTs included in our study, the DIGIT-HF and DIG trials contributed most substantially to the overall findings. The DIG trial, published in 1997, concluded that administration of digoxin to the then-available GDMT only reduced rate of HF hospitalization while not all-cause mortality. Subsequent *post-hoc* analysis of the DIG trial and meta-analysis also revealed that patients with lower serum digoxin concentrations demonstrated greater clinical benefits compared to those with higher concentrations (9, 10).

The DIGIT-HF trial is the only RCT investigating cardiac glycosides for heart failure within the context of contemporary GDMT. The results demonstrated that the treatment with digitoxin led to a lower combined risk of death from any cause or hospital admission for worsening heart failure than placebo among patients with HFrEF. In the DIGIT-HF trial, a titration strategy guided by therapeutic drug monitoring was employed, which may be the key reason for its more pronounced benefits. Although the pooled analysis in our study did not demonstrate a mortality benefit from cardiac glycosides in HFrEF patients, the findings suggest that we need to reassess the role of these agents, particularly digitoxin, in the management of HFrEF.

Although digoxin and digitoxin share similar pharmacodynamic properties, digitoxin exhibits distinct pharmacokinetic advantages. The lipophilic nature of digitoxin contributes to its higher intestinal absorption and stronger serum protein binding (5, 11). Digoxin is eliminated predominantly by the kidneys, whereas digitoxin undergoes substantial enterohepatic circulation, enabling effective clearance even in cases of severe renal impairment and yielding more stable plasma concentrations (5). However, our indirect comparison failed to suggest a potential advantage of digitoxin over digoxin, which may be attributed to the small sample size of the available studies. Of the 6 studies included, only one (DIGIT-HF) evaluated digitoxin as an intervention, involving 613 patients. To date, no randomized controlled trials (RCTs) or observational studies have directly compared the efficacy of digoxin and digitoxin in improving clinical outcomes. Therefore, the current findings should be regarded as hypothesis-generating.

In the absence of head-to-head RCTs comparing digitoxin and digoxin, we performed an exploratory network meta-analysis to enable an indirect comparison between the two agents. It is noteworthy that our indirect comparison did not account for the evolution of GDMT. The nearly 30-year span from the publication of the DIG trial in 1997 to the anticipated publication of DIGIT-HF in 2025 represents a substantial period during which foundational heart failure treatments have advanced significantly. Over this period, GDMT for heart failure has undergone substantial changes. The introduction of currently recommended agents such as angiotensin receptor neprilysin inhibitors (ARNI) (12), sodium-glucose co-transporter 2 inhibitor, (SGLT2i) (13), and guanylate cyclase stimulators (14) has significantly improved patient outcomes. Furthermore, device therapies like ICDs and CRT have become widely adopted, substantially reducing mortality (15, 16). According to data from Swedish National Patient Register, these advances have lowered the annual heart failure mortality rate from 33.4 to 23.8 per 100 000 individuals between 1997 and 2022 (17). These therapeutic interventions have markedly improved the prognosis of patients with heart failure with HFrEF. On the other hand, these advances may have statistically attenuated the apparent clinical benefits of cardiac glycosides. The evolution of GDMT undoubtedly influenced the results of the indirect comparison in our study; therefore, these findings should be interpreted with caution.

Additionally, the ongoing DECISION trial (18), an RCT conducted within the framework of contemporary GDMT, is designed to evaluate the effect of low-dose digoxin on mortality in HFrEF patients. Upon its completion, the results will provide higher-quality evidence, thereby allowing for a more definitive comparison of the clinical benefits between digitoxin and digoxin.

### 4.3 Comparison with previous studies

The meta-analysis by Vamos (19) suggested that digoxin may be associated with an increased risk of mortality among patients with heart failure. Their results are not consistent with our results. In contrast to our study, which exclusively included RCTs, their analysis incorporated observational studies in the pooled data. The outcome of increased mortality appears to be largely driven by data from observational studies, which are susceptible to unmeasured confounding, along with findings from specific subgroup analyses (20). It is noteworthy that, according to current clinical guidelines, in patients with symptomatic HFrEF despite GDMT (or who are unable to tolerate GDMT), digoxin might be considered to decrease hospitalizations for HF (21). The use of digoxin is often associated with patients who have more severe clinical manifestations, poorer cardiac function, and a greater burden of comorbidities. Even when observational studies attempt to adjust for these factors using Cox proportional hazards models or propensity score matching, they cannot fully eliminate the potential influence of such confounding variables. This is precisely why our study did not consider including observational studies. In 2015, Ziff published another systematic review to assess the risks associated with digoxin use (22), which stratified its analysis by study design. Its results demonstrated no association between digoxin use and increased all-cause mortality when only RCTs were considered. While this finding aligns with our results, the analysis by Ziff included patients with heart failure with preserved ejection fraction (HFpEF). Given the distinct pathophysiological mechanisms underlying HFpEF and HFrEF and considering that the pharmacological action of cardiac glycosides targets the impaired systolic function characteristic of HFrEF, we excluded patients with HFpEF from our study population.

### 4.4 Limitation

Our trial has limitations. First, our analysis did not incorporate observational studies. While numerous recent clinical studies conducted within the contemporary GDMT framework have been published—and including observational data might further clarify the benefits of cardiac glycosides in this context—their use in real-world cohorts is inherently associated with more severe clinical profiles. Statistical adjustments in observational research cannot fully eliminate the confounding effects of these factors. Second, our study is a study-level meta-analysis. As many of the included trials are relatively dated, patient-level data were unavailable, precluding subgroup analyses to identify specific patient populations that might derive greater benefit. Third, the included studies exhibited a wide range of follow-up durations, during which significant evolution has occurred in the foundational treatment of heart failure. This temporal disparity may introduce a certain degree of bias to the overall results.

### 4.5 Conclusion and prospect

Based on current evidence, the use of cardiac glycosides in patients with HFrEF does not reduce all-cause mortality but does lower the risk of heart failure hospitalization. Digitoxin may not hold a potential advantage over digoxin in improving prognosis of HFrEF patients. However, given the potential for bias, RCTs with larger sample size or head-to-head RCTs are warranted to provide more robust evidence.

## Data Availability

The original contributions presented in the study are included in the article/Supplementary Material, further inquiries can be directed to the corresponding authors.
