# Supplementary material for: Cardiac glycosides in patients with heart failure and reduced ejection fraction: a systematic review and meta-analysis
Source: Front Cardiovasc Med. 2026 Mar 13;13:1746467. doi: 10.3389/fcvm.2026.1746467 (PMC13021435; doi:10.3389/fcvm.2026.1746467)
Supplement: Supplementary file 1 [file Datasheet1.docx]

Supplementary Material

# Supplementary Tables

## Table S1

| Table S1. Search strategy. | |
| --- | --- |
| Database | Search strategy |
| PubMed  (n=306) | (((heart failure with reduced ejection fraction [Title/Abstract]) OR (HFrEF [Title/Abstract]) OR (heart failure and reduced ejection fraction [Title/Abstract]) OR (congestive heart failure[Title/Abstract]) OR (systolic heart failure [Title/Abstract]))  AND ((digoxin [Title/Abstract]) OR (digitoxin [Title/Abstract]) OR (digitalis [Title/Abstract]) OR (cardiac glycoside [Title/Abstract]) OR (cardiac glycosides [Title/Abstract]))  AND ((Mortality[Title/Abstract] ) OR (Death[Title/Abstract] ) OR (Rehospitalization[Title/Abstract] ) OR (Hospitalization[Title/Abstract] ) OR (readmission[Title/Abstract])))  NOT ((Review[Filter]) OR (Meta-Analysis [Filter]) OR(Case Reports [Filter]) OR (Books and Documents [Filter]) OR (Editorial[Filter])) |
| EMBASE  (n=497) | (((heart failure with reduced ejection fraction):ti,ab,kw OR (HFrEF):ti,ab,kw OR (heart failure and reduced ejection fraction):ti,ab,kw OR (congestive heart failure):ti,ab,kw OR (systolic heart failure):ti,ab,kw)  AND ((digoxin):ti,ab,kw OR digitoxin):ti,ab,kw OR (digitalis):ti,ab,kw OR (cardiac glycoside):ti,ab,kw OR (cardiac glycosides):ti,ab,kw)  AND ((Mortality):ti,ab,kw OR (Death):ti,ab,kw OR (Rehospitalization):ti,ab,kw OR (Hospitalization):ti,ab,kw OR (readmission):ti,ab,kw) AND ('Article'/it OR 'Article in Press'/it OR 'Conference Paper'/it)) NOT ('case report'/de OR 'nonhuman'/de OR 'meta analysis'/de OR 'systematic review'/de) |
| Cochrane Library (n=215) | ((heart failure with reduced ejection fraction):ti,ab,kw OR (HFrEF):ti,ab,kw OR (heart failure and reduced ejection fraction):ti,ab,kw OR (congestive heart failure):ti,ab,kw OR (systolic heart failure):ti,ab,kw)  AND ((digoxin):ti,ab,kw OR (digitoxin):ti,ab,kw OR (digitalis):ti,ab,kw OR (cardiac glycoside):ti,ab,kw OR (cardiac glycosides):ti,ab,kw)  AND ((Mortality):ti,ab,kw OR (Death):ti,ab,kw OR (Rehospitalization):ti,ab,kw OR (Hospitalization):ti,ab,kw OR (readmission):ti,ab,kw) |
| Web of Science (n=163) | #1 TS=( heart failure with reduced ejection fraction OR HFrEF OR heart failure and reduced ejection fraction OR congestive heart failure  OR systolic heart failure)  #2 TS=(cardiac glycoside OR cardiac glycosides OR digitalis OR digitoxin OR digoxin)  #3 TS=(Mortality OR Death OR Rehospitalization OR Hospitalization  OR Readmission)  #4 DT==("ARTICLE")  #5 DT==("REVIEW" OR "MEETING" OR "CASE REPORT" OR "EDITORIAL MATERIAL" OR "REFERENCE MATERIAL" OR "ABSTRACT" OR "LETTER" OR "NEWS")  #6 #1 AND #2 AND #3 AND #4 NOT #5 |
|  | |

# Supplementary Figures


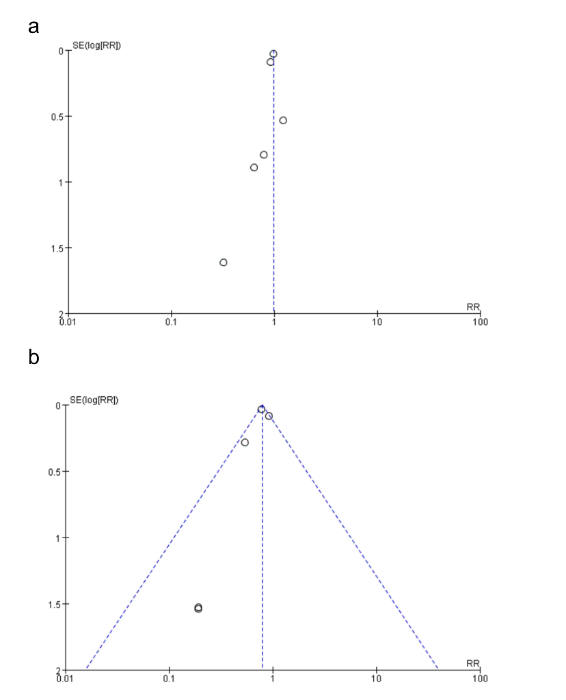


**Supplementary Figure S1.** Funnel Plot (a) all cause mortality (b) heart failure rehospitalization.


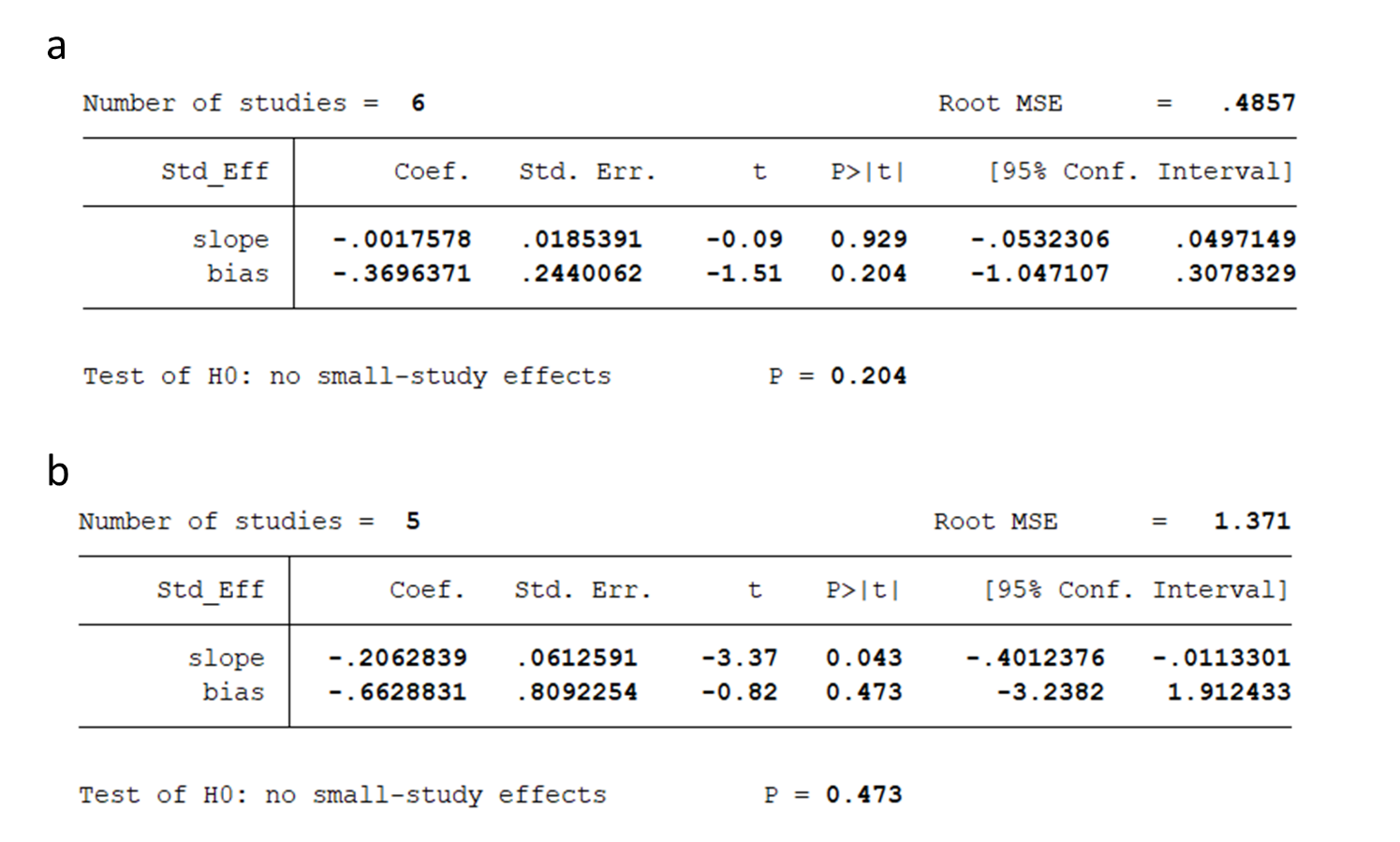


**Supplementary Figure S2.** STATA publication bias analysis.
